# Case of Fistulous Opening between the Urethra and Vagina, Successfully Treated. Case of Incontinence of Urine Cured by Operation

**Published:** 1830-10

**Authors:** Samuel Hobart

**Affiliations:** Cork.


THE LONDON
### Medical and Physical Journal.

No. 380, VOL. LXIV.] OCTOBER 1830. [No. 52, *New Series*.

For many fortunate discoveries in medicine, and for the detection of numerous errors, the world is indebted to the rapid circulation of Monthly Journals; and there never existed any work to which the Faculty, in Europe and America, were under deeper obligations than to the *Medical and Physical Journal of London*, now forming a long but invaluable series.—RUSH.

### ORIGINAL PAPERS, AND CASES,

### OBTAINED FROM PUBLIC INSTITUTIONS AND OTHER AUTHENTIC SOURCES.

### FISTULOUS OPENINGS.

*Case of Fistulous Opening between the Urethra and Vagina, successfully treated. Case of Incontinence of Urine cured by Operation.* By SAMUEL HOBART, Esq. Cork. (Communicated by Mr. BRODIE.)

THE communication which I submitted to you in the autumn of 1825, upon Fistulous Openings between the Urethra and Vagina, having been inserted in the London Medical and Physical Journal of December 1825, accompanied by a plate of the instruments used upon that occasion, I am not without the most sanguine hopes that, in other hands, the treatment has been equally successful.

It is remarkable that I should have had another opportunity of proving the advantages of the above operation, as Catherine Mahony, the case alluded to, having again become pregnant, a similar calamity occurred, and a similar mode of treatment has been productive of an equally fortunate result as in the former instance; the detail of which has been amply given in the London Medical and Physical Journal of December, 1825.

I feel, therefore, I should be deficient in my duty to the profession, if I did not request you again to receive my communication; as I fear that similar cases must be much more frequent than is imagined.

It has long been supposed by physiologists that the bladder alone was engaged in the expulsion of the urine.

The following case, however, is so important in a practical point of view, as proving the muscular powers of the female urethra, that I think it cannot better be brought forward to the notice of the profession than in a paper devoted entirely to the urinary organs.

CASE. Mary Connor, a respectable young woman, æt. twenty, labouring under incontinence of urine, consulted me some time since, in the expectation of receiving some relief from symptoms which rendered her existence miserable. She gave me the following statement of her case: That she had fallen down stairs about three years before, and had injured her back in the fall, from which period, up to the time of her placing herself under my care, she gradually lost all command over the contents of the bladder: she had previously taken the advice of many professional men in Cork and elsewhere, without receiving any benefit, and at the time above alluded to, suffered extremely from excoriation of the labia, and inside of the lower extremities, from the constant flow of urine. The opinion entertained of her case was, that the symptoms were caused by paralysis of the neck of the bladder, produced by the fall, and that little or nothing could be done for her relief.

On examination, I found the urethra so much enlarged (so that, unless the power of retaining the urine depended entirely on the sphincter vesicæ, no check could be given whatsoever to its constant flow,) as to admit with facility a full-sized rectum bougie. The sides of the canal lay quite loose and flabby, appearing to have entirely lost the power of contractility. It appeared to me that a certain degree of harmony should exist between the bladder and urethra, to produce the natural control over the contents of the former viscus: with this view I proposed diminishing the diameter of the canal, by removing a portion of its substance; but my professional brethren in attendance would not consent to my suggestion, as they were of opinion that it would not be productive of success: it was therefore abandoned until other means should be tried. Every plan that could be devised, and every medicine that could be thought of, were employed, but without producing the slightest improvement. I, then, a second time proposed my former plan of treatment, which was agreed upon, and the operation was performed in the following manner, in the presence of my friends Drs. PITCAIRN and BULL.

Having placed her on her knees on a table, with her head downwards, and dilated the vagina with Weis's speculum, I introduced the index finger of my left hand into the urethra as far as the neck of the bladder, and having passed a sharp-pointed bistoury to the end of my finger, then raising its point, and cutting through the side of the urethra, so as to form one cavity of that canal and the vagina; I removed a portion of the urethra, the entire length of the canal, and of a triangular shape, with a knife-edged scissors. The lips of the wound were then brought together by means of pins, and secured by the interrupted suture.

The pins were all removed at the end of eight days, leaving the parts perfectly united, and the woman completely restored, having entirely recovered the perfect control over the discharge of urine. The urethra at this time has to all appearance recovered its natural tone and size.

That this case throws an important light upon the functions of the urethra, is but too evident. A very intelligent teacher of anatomy, to whom the detail was submitted, looked upon it as a forlorn hope, and that a "dripping" urethra would be her portion for the remainder of her life. I confess, however, I was not oversanguine myself in the result; but I felt so much interest in a case that I had anxiously watched for three years, that I determined to make the experiment, and the consequence has been not only the restoration of a young woman to the enjoyments of all the comforts of life, but also to having enabled her to become a wife, and possibly in due time a mother.

---

